# Synchronous imaging of pelvic geometry and muscle morphometry: a pilot study of pelvic retroversion using upright MRI

**DOI:** 10.1038/s41598-021-99305-w

**Published:** 2021-10-11

**Authors:** Noor Shaikh, Honglin Zhang, Stephen H. M. Brown, Hamza Lari, Oliver Lasry, John Street, David R. Wilson, Thomas Oxland

**Affiliations:** 1 School of Biomedical Engineering, University of British Columbia, Vancouver, Canada; 2 ICORD, University of British Columbia, Vancouver, Canada; 3 Department of Mechanical Engineering, University of British Columbia, Vancouver, Canada; 4 Centre for Hip Health and Mobility, University of British Columbia, Vancouver, Canada; 5 Department of Human Health and Nutritional Sciences, University of Guelph, Guelph, Canada; 6 Department of Radiology, Vancouver General Hospital, Vancouver, Canada; 7 Department of Orthopaedics, University of British Columbia, Vancouver, Canada; 8 University of British Columbia, ICORD, Blusson Spinal Cord Centre at VGH, 3rd Floor, 818 West 10th Avenue, Vancouver, BC V5Z 1M9 Canada

**Keywords:** Musculoskeletal system, Magnetic resonance imaging

## Abstract

This study investigated feasibility of imaging lumbopelvic musculature and geometry in tandem using upright magnetic resonance imaging (MRI) in asymptomatic adults, and explored the effect of pelvic retroversion on lumbopelvic musculature and geometry. Six asymptomatic volunteers were imaged (0.5 T upright MRI) in 4 postures: standing, standing pelvic retroversion, standing 30° flexion, and supine. Measures included muscle morphometry [cross-sectional area (CSA), circularity, radius, and angle] of the gluteus and iliopsoas, and pelvic geometry [pelvic tilt (PT), pelvic incidence (PI), sacral slope (SS), L3–S1 lumbar lordosis (LL)] L3-coccyx. With four volunteers repeating postures, and three raters assessing repeatability, there was generally good repeatability [ICC(3,1) 0.80–0.97]. Retroversion had level dependent effects on muscle measures, for example gluteus CSA and circularity increased (up to 22%). Retroversion increased PT, decreased SS, and decreased L3–S1 LL, but did not affect PI. Gluteus CSA and circularity also had level-specific correlations with PT, SS, and L3–S1 LL. Overall, upright MRI of the lumbopelvic musculature is feasible with good reproducibility, and the morphometry of the involved muscles significantly changes with posture. This finding has the potential to be used for clinical consideration in designing and performing future studies with greater number of healthy subjects and patients.

## Introduction

Up to 60% of aging adults are affected by some form of adult spinal deformity (ASD)^1^. ASD includes scoliosis, sagittal malalignment, kyphosis, and spondylolisthesis^1^. In particular, loss of lumbar lordosis (LL), either iatrogenic or due to progressive degeneration, especially when combined with increased thoracic kyphosis (thoracic spine angle > 40°), which affects 20–40% of adults over 60 years^2^, can lead to positive sagittal balance disorder. Positive sagittal balance is characterized by an anterior shift of the body’s C7 plumb line relative to the posterosuperior corner of S1 (quantified by sagittal vertical axis (SVA)), and has been identified as the most reliable radiographic predictor of clinical health status^3,4^. Contributing risk factors include vertebral fractures, disc degeneration, decreased mobility, proprioceptive deficits, and genetic basis. More recently, the role of spinal muscle weakness and dysfunction have been emphasized^5,6^.

In response to positive sagittal balance, patients use musculoskeletal compensatory mechanisms to regain sagittal balance (SVA < 5 cm) and horizontal gaze^1^. One of the first, and most effective, mechanisms recruited is pelvic retroversion. Others include segmental lumbar hyperlordosis, segmental retrolisthesis, thoracic hypokyphosis, knee flexion, and cervical hyperlordosis^7–10^. Such compensatory mechanisms require higher energy expenditure due to muscle activation to maintain these postures, which can lead to patient fatigue, discomfort, disability, and poor clinical outcomes^11^. Long term, increasing deformity or insufficient muscle strength and endurance can make these mechanisms inadequate to maintain alignment. Though spinal muscle dysfunction has been identified to play a critical role in sagittal balance disorder, the upright lumbopelvic muscle morphometry and the interactions with the bony pelvic geometry have not been previously studied.

Medical imaging has been used extensively to study lumbopelvic musculature and geometry. This includes X-ray^8,10^, ultrasound^12,13^, computed tomography (CT)^14^, and magnetic resonance imaging (MRI)^15–20^. Though X-ray can provide upright and postural bony geometry, it lacks soft tissue information^8–10,21–24^. Additionally, though ultrasound can be performed upright, it lacks imaging depth and resolution^12,13^. CT and MRI can provide 3-dimensional detail but typically lack upright functionality^14,15,17–20^. MRI however, though primarily supine, has also shown some promising upright and postural results for soft tissue measurements. In one study, upright MRI was used to demonstrate that head-neck position significantly influences the cross-sectional area (CSA) and position of neck muscles^16^. There were also some neck muscle CSA trends which varied compared to trends from prior supine MRI studies which supports the importance of considering postural and upright imaging, rather than just supine^16^. Our group has recently shown lumbar muscle and level specific trends with upright and seated postures when considering the multifidus/erector spinae and psoas major muscles^25^ and described guidelines for the quantification of thoracic spine musculature^26^. Additionally, in the pelvic region, studies have considered compressive deformation of the gluteus maximus during seated and tilting postures^27,28^.

Though there is growing interest in the lumbopelvic muscles and geometry in standing and compensatory postures, there is a gap in the literature in understanding the underlying mechanisms of such compensatory changes and the associated muscle and bony geometry interactions. As pelvic retroversion, by increasing pelvic tilt, is typically an initial compensatory step, groundwork in studying both the lumbopelvic musculature and geometry synchronously in upright and retroverted postures first in asymptomatic individuals would support subsequent patient studies to inform future treatment and mitigation of ASD. To our knowledge, synchronous study has not previously been done, and it is also key to determine repeatability of image segmentation of both lumbopelvic muscle morphometry and bony geometry in tandem before moving to a patient population.

Therefore, the primary objective of this study was to determine feasibility and repeatability of imaging lumbopelvic musculature and geometry in tandem using upright magnetic resonance imaging (MRI) in asymptomatic adults. A secondary objective was to explore the effect of pelvic retroversion on the lumbopelvic musculature muscle [cross-sectional area, circularity and position (radius, angle)] and geometry (pelvic tilt, pelvic incidence, sacral slope, L3–S1 lumbar lordosis).

## Methods

### Study participants

Six asymptomatic volunteers were recruited (4 females, 2 males; median and range of indicators: age 27 (24–50) years, height 172 (160–185) cm, mass 66 (57–80) kg, BMI 23 (22–25), no spine or pelvic conditions). This study was conducted with approval from the University of British Columbia’s Clinical Research Ethics Board (CREB # H10-00942). Volunteers verbally confirmed they had no previous spinopelvic conditions. All methods were performed in accordance with relevant guidelines and regulations, and all volunteers provided oral and written informed consent to participate in the study.

### Image acquisition

**Figure 1:**
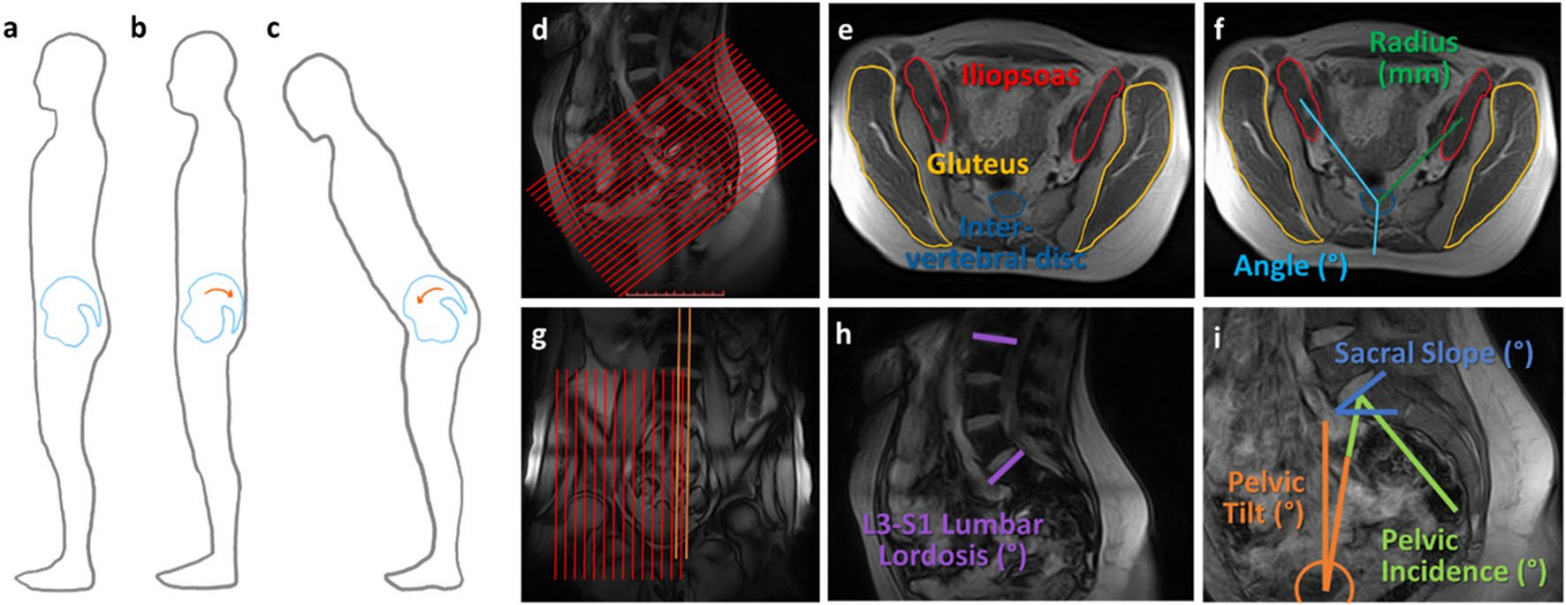
Diagram of postures, image slice orientation, and image analysis measurements. (**a**) Standing posture, (**b**) Standing with pelvic retroversion (retroversion) posture, (**c**) Standing with 30° flexion from the hip (flexion) posture. (**d**) For muscle morphometry, an oblique image stack was aligned to the sacral endplate and covered midpoint of L5 to the coccyx. (**e**) Muscle cross-sectional area (CSA) boundaries were outlined for the gluteus (yellow) and iliopsoas (red), with the intervertebral disc also outlined (blue). Circularity and centroid of the CSA were automatically determined in Image J. (**f**) Position measurements of radius (mm) (dark green) as distance between centroid of the muscle to centroid of the intervertebral disc, and angle (degrees) (blue) measured relative to the line running posteriorly from the intervertebral disc centroid to the spinous process. (**g**) For pelvic geometry, a sagittal scan was aligned to the midline of the spinal column covering L3 to S1 (orange), and a sagittal stack covered the femoral head and midline of the spinal column on the right (red). (**h**) Measurement of L3–S1 lumbar lordosis (purple) as the angle between the L3–S1 superior endplates. (**i**) Measurements of pelvic tilt (orange), pelvic incidence (green), and sacral slope (blue).

The volunteers were imaged within the 56 cm gap of a 0.5 T vertical open MRI scanner (MROpen, Paramed, Genoa, Italy) with a one channel loop coil that went around their hips. For muscle morphometry measures, the sequence used was a T1-weighted Spin Echo with an oblique-axial stack aligned to the sacral endplate and covering the middle of the L5 vertebral body to the coccyx (Fig. 1d, imaging parameters Table 1). For geometry measures, the sequences included a T1-weighted Gradient Echo with a sagittal slice at the midline of the spinal column covering L3–S1, and a T1-weighted Spin Echo with a sagittal stack covering the femoral head to the midline of the spinal column on the right side (Fig. 1g, imaging parameters Table 1). Each volunteer was scanned in 4 postures: supine, standing, standing with pelvic retroversion (retroversion), and standing with 30° flexion from the hip (flexion) (Fig. 1a–c). For pelvic retroversion, the volunteers were asked to retrovert or tuck their pelvic maximally. The volunteers were asked to hold all postures naturally with bars and foam around them to minimize movement. The bars and foam only served to prevent volunteers from swaying rather than to support load and volunteers were instructed not to rely on the bars for support and rather as indicators for where to hold postures. For posture repeatability, four volunteers were re-scanned in all upright postures, after being removed and repositioned.

**Table 1:** Imaging sequence parameters.

Region/anatomy	Sequence	Alignment	TR/TE (ms)	FOV (cm)	Scan matrix	Slice thickness (mm)	NEX	Scan time (s)
Muscle morphometry	T1-weighted Spin Echo	Oblique-axial stack, L5-coccyx	586/12	40	224 × 160	7, 0.7 gap	1	114
Lumbar geometry	T1-weighted Gradient Echo	Sagittal slice, midline spinal column, L3–S1	44/7	35	192 × 128	15, 1 gap	1	14
Pelvic geometry	T1-weighted Spin Echo	Sagittal stack, femoral head to midline spinal column on right	323/10	24	224 × 192	10, 1 gap	1	139

### Image analysis

Image analysis for muscle morphometry used ImageJ (Version 1.52; National Institutes of Health, USA). On the oblique scans, levels L5/S1, S1/S2, and S4/S5 were identified as the slice bisecting the intervertebral disc, and the level of maximum femoral head area (max FH) was identified as the slice bisecting the maximum femoral head cross-sectional area, by referencing the sagittal images. Measurements of two-dimensional muscle cross-sectional area (CSA), circularity, and position (angle and radius describing the position of the muscle centroid relative to the centroid of the disc) were taken (Fig. 1e,f) on the gluteus (maximus, medius, minimus combined) and iliopsoas (iliacus and psoas major at L5/S1) muscles (Table 2)^16^. As the demarcation between muscles was not always evident, the gluteus maximus, medius, and minimus muscles were combined, as well as the iliacus and psoas major. Muscle CSA (mm^2^) was determined by manually tracing the muscle boundary. The muscle circularity and centroid were automatically determined in ImageJ. Muscle circularity was calculated as, $circularity=\frac{4*\pi *area}{{(perimeter)}^{2}}$ (1 indicates a circle, approaching 0 indicates an elongated polygon). Muscle centroid was defined as the geometric center. Muscle angle (degrees) was measured between the line connecting the muscle centroid to the intervertebral disc centroid and the line connecting the intervertebral disc (or sacral body) centroid to the spinous process. Muscle radius (mm) was measured as the distance from the muscle centroid to the intervertebral disc (or sacral body) centroid (Fig. 1f)^16^. Right and left values were averaged, except in correlation and repeatability tests where right were defined as volunteer right, and left were defined as volunteer left.

**Table 2:**
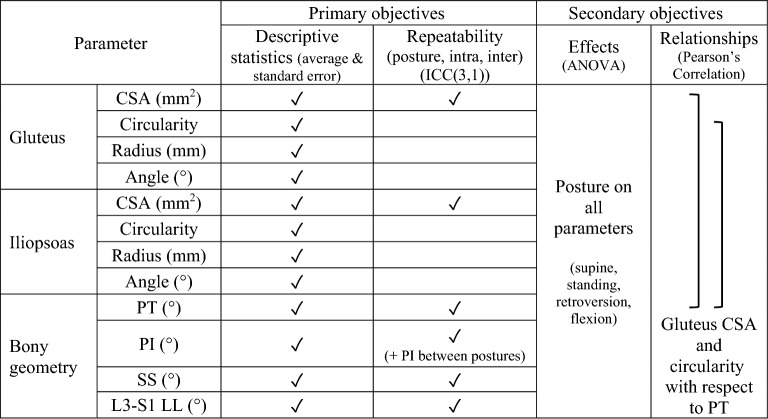
Summary of measured parameters and investigated relationships.

Image analysis for geometry used RadiAnt DICOM Viewer (Version 5.5.1; Medixant, Poland). On the sagittal slice, L3–S1 lumbar lordosis (L3–S1 LL) was the angle between the L3–S1 superior endplates (Fig. 1h). On the sagittal stack pelvic tilt (PT), pelvic incidence (PI), and sacral slope (SS) were determined (Fig. 1i). By superimposing the segmentation of the maximal femoral head area onto the image of the spinal column midline, PT was the angle between a line from the center of the femoral head to the mid-point of the sacral endplate and the vertical. PI was the angle between the line perpendicular to the midpoint of the sacral endplate and the line from the center of the femoral head to the mid-point of the sacral endplate. SS was the angle of the sacral endplate relative to the horizontal (Table 2)^29^.

One rater (biomedical engineer) segmented all images (144 for initial imaging, 78 for posture repeatability). Two raters (1 musculoskeletal radiologist, 1 spine surgery fellow) segmented a subset of images for inter- and intra-rater repeatability (102 scans). Segmentation was performed individually after reviewing muscle anatomy and repeats were performed after two weeks. All segmentations were reviewed for quality control by the primary author with no corrections of segmentation needed.

### Statistical analysis

Statistical analysis used Statistica (Version 13, Copyright 1984–2017, TIBCO Software Inc.). For the primary objective, the average and standard error was determined for each parameter and for each muscle (Table 2). Repeatability was assessed on muscle CSA and bony geometry measures using intraclass correlation coefficients [ICC(3,1)]. Posture repeatability compared measures in the re-scanned volunteers. Intra-rater repeatability was assessed on measures by the same rater and inter-rater repeatability considered all three raters. PI between postures for each volunteer was also considered. ICCs were interpreted as: < 0.69 poor, 0.70–0.79 fair, 0.80–0.89 good, and 0.90–1.00 excellent^30^. Mean absolute difference (MAD) of muscle CSA was also calculated between posture repetitions and between volunteers.

For the secondary objective, for each muscle, the effect of posture and pelvic/vertebral level on muscle CSA, circularity, radius, and angle was evaluated with a 2-way repeated measures analysis of variance (ANOVA, *P* < 0.05) (Table 2). A 1-way repeated measures ANOVA (*P* < 0.05) was used to evaluate the effect of posture on pelvic geometric parameters. Normality and sphericity were confirmed, and post-hoc analyses were completed using Neuman-Keuls. The relationship between changing muscle CSA and circularity of the gluteus, and lumbopelvic geometric measures of PT, SS, and L3–S1 LL were evaluated using Pearson’s correlation coefficient (*P* < 0.05). For correlations, to minimize bias of body size, muscle CSA was normalized by maximum femoral head cross-sectional area, which through previous studies has been shown to be related to an individual’s body size^31–33^. Pelvic geometric parameters (PT, SS, LL) were normalized by supine values of the respective parameter.

## Results

### Primary objective: descriptive statistics

For average muscle parameters across all postures and levels, CSA was 2920–7466 mm^2^ for the gluteus, and 539–3103 mm^2^ for the iliopsoas (Table 3). Muscle circularity was 0.35–0.54 for the gluteus, and 0.52–0.70 for the iliopsoas (Table 3). Muscle radius was 75–114 mm for the gluteus, and 75–162 mm for the iliopsoas (Table 3). Muscle angle was 97°–117° for the gluteus, and 110°–148° for the iliopsoas (Table 3).

**Table 3:** Average muscle parameter values.

Parameter		L5/S1		S1/S2		Max FH		S4/S5	
		Average range	Standard error range	Average range	Standard error range	Average range	Standard error range	Average range	Standard error range
Gluteus	CSA (mm^2^)	2920–3767	195–385	5025–6120	388–536	6413–7466	566–602	4822–5720	330–623
	Circularity	0.35–0.42	0.01–0.02	0.45–0.54	0.01–0.02	0.37–0.47	0.01–0.02	0.40–0.53	0.02–0.03
	Radius (mm)	108–111	0.7–1.9	112–114	2.3–3.4	102–109	3.0–4.5	75–105	2.3–9.5
	Angle (°)	97–101	1.5–2.4	98–110	1.8–7.3	111–116	0.9–2.22	101–111	1.0–2.3
Iliopsoas	CSA (mm^2^)	2559–3103	347–189	2235–1959	256–125	603–718	60–81	538–564	57–101
	Circularity	0.53–0.60	0.01–0.02	0.52–0.58	0.01–0.04	0.60–0.69	0.01–0.03	0.65–0.71	0.02–0.04
	Radius (mm)	75–79	1.8–2.8	99–108	4.7–9.11	157–162	2.8–4.5	147–160	2.7–12.4
	Angle (°)	110–117	1.6–3.7	130–139	2.0–3.9	145–148	0.3–0.5	135–145	0.46–10.4

For average bony geometry measures across all postures, PT was 9°–24°, SS was 34°–53°, PI was 54°–57°, L3–S1 LL was 28°–53° (Table 4).

**Table 4:** Average bony geometry parameter values.

Parameter		Average range	Standard error range
Geometry	PT (°)	9–24	2–4
	PI (°)	34–35	1–3
	SS (°)	54–57	2–3
	L3–S1 LL (°)	28–53	3–6

### Primary objective: repeatability

The average posture repeatability for muscle CSA in the 4 rescanned volunteers was 0.90 [ICC(3,1)] (ranges 0.86–0.94) for the gluteus, and 0.93 (range 0.90–0.97) for the iliopsoas (Supplementary Material Table 1). The mean absolute difference for repeats of the same posture ranged from 490 to 759 mm^2^ for the gluteus, and from 191 to 515 mm^2^ for the iliopsoas. For comparison, the mean absolute difference between volunteers was also determined. It ranged from 1350 to 1541 mm^2^ for the gluteus, and from 543 to 677 mm^2^ for the iliopsoas.

The average posture repeatability for all pelvic geometry in the 4 rescanned volunteers was 0.97 (range 0.94–0.99) (Supplementary Material Table 2). The mean absolute difference for repeats of the same posture was 1°–6°. For comparison, the mean absolute difference between volunteers was 9°–13°. Across postures, PI repeatability was 0.85–0.92.

The average intra-rater repeatability for muscle CSA for raters across all levels and sides was 0.91 (range 0.82–0.99) for the gluteus, and 0.84 (range 0.62–0.95) for the iliopsoas (Supplementary Material Table 3). For pelvic geometry, the average intra-rater repeatability for raters across all parameters was 0.92 (range 0.76–0.99) (Supplementary Material Table 4).

The average inter-rater repeatability for muscle CSA for raters across all levels and sides was 0.88 (range 0.76–0.96) for the gluteus, and 0.71 (range 0.54–0.89) for the iliopsoas (Supplementary Material Table 5). For pelvic geometry, the average inter-rater repeatability for raters across all parameters was 0.87(range 0.79–0.94) (Supplementary Material Table 6).

### Secondary objective: muscle CSA, circularity, radius and angle

**Figure 2:**
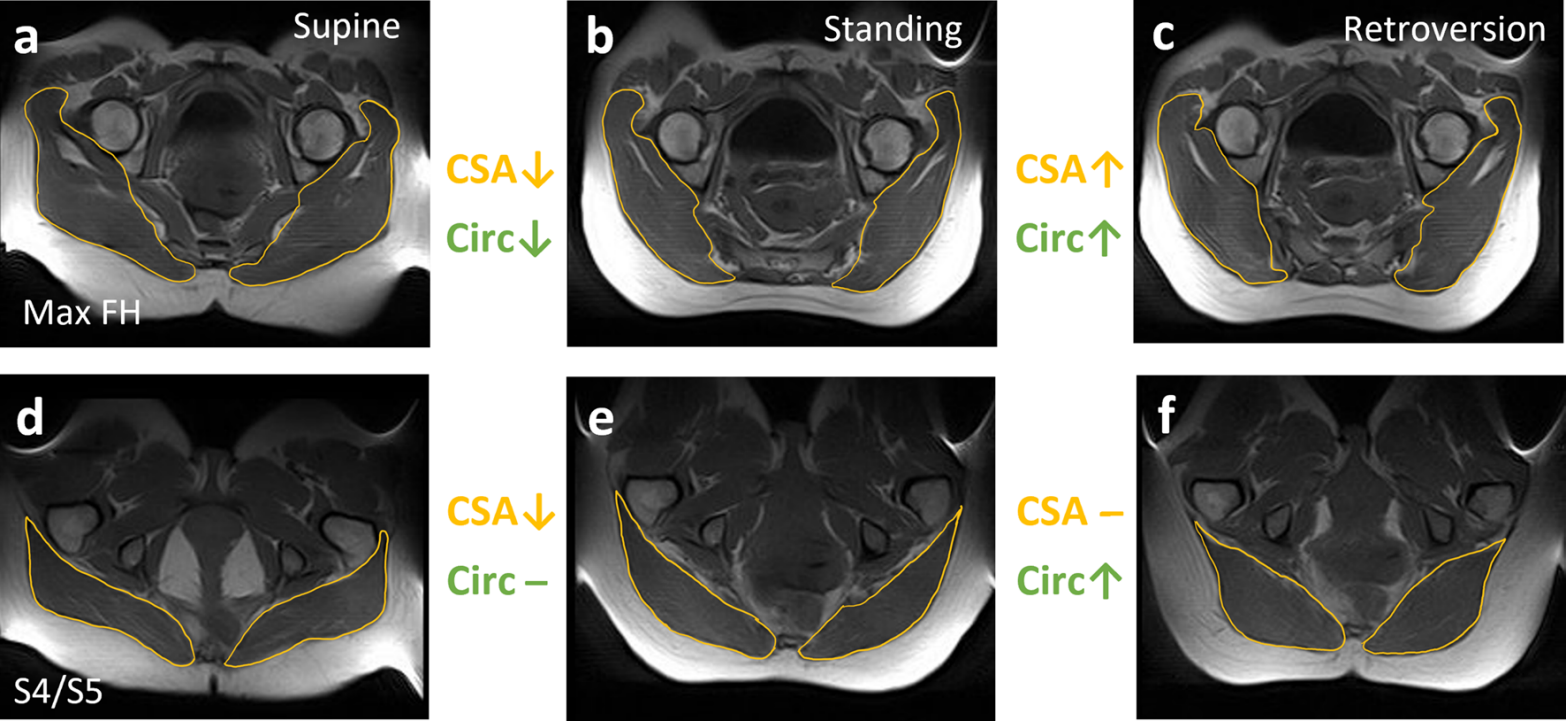
Example changes in gluteus CSA (yellow) and circularity (Circ, green) with posture (↓ = decrease, ↑ = increase, – = no change). At max FH for both CSA and circularity, there was up to a 15% decrease from supine to standing (**a**, **b**) and up to a 22% increase from standing or flexion to retroversion (b to c). At S4/S5, from supine to standing though CSA decreased by 16%, there was no change in circularity (**d**, **e**). Additionally, from standing or flexion to retroversion, though there was no change in CSA, circularity increased up to 24% (**e**, **f**).

**Figure 3:**
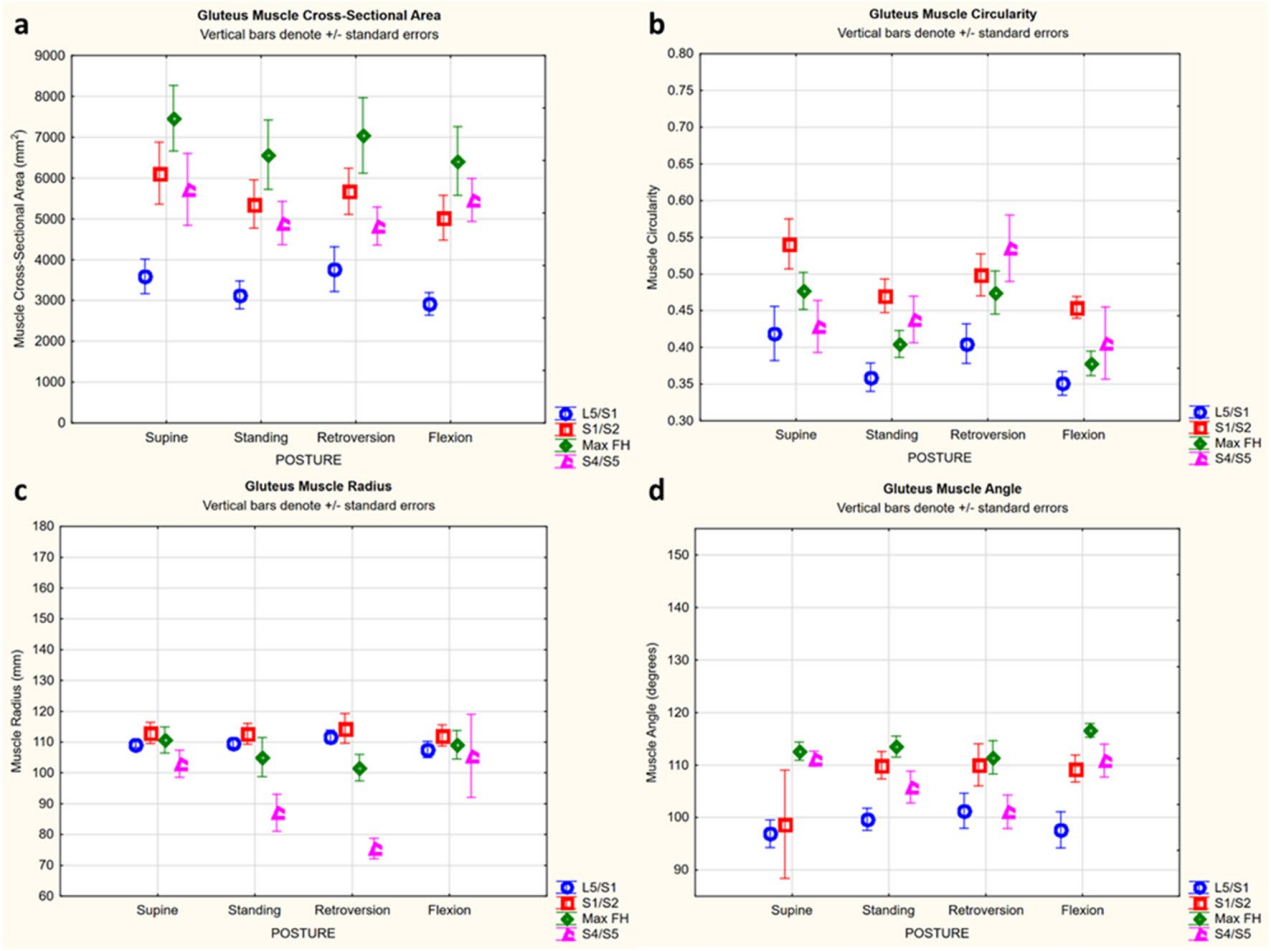
Mean (± SEM) gluteus parameter for each posture shown by level. (**a**) CSA: there was a significant effect and interaction of posture and level (Newman–Keuls significant: supine to standing at max FH, 13% decrease; flexion to retroversion at L5/S1, 22% increase; supine to standing at S4/S5, 16% decrease). (**b**) Circularity: there was a significant effect and interaction of posture and level (Newman–Keuls significant: supine to standing at S1/S2, up to 15% decrease; flexion to retroversion at max FH, up to 20% increase; standing to retroversion at S4/S5, 18% increase; flexion to retroversion at S4/S5, 24% increase). (**c**) Radius: There was a significant effect and interaction of posture and level (Newman–Keuls significant at S4/S5: supine to standing, 18% decrease; supine to retroversion, 36% decrease; standing to retroversion, 15% decrease; flexion to standing, 21% decrease; flexion to retroversion, 40% decrease). (**d**) Angle: there was a significant effect and interaction of posture and level (Newman–Keuls significant: supine to standing, 10% increase at S1/S2; supine to flexion, 10% increase at S1/S2; supine to retroversion, 10% increase at S1/S2; supine to retroversion at S4/S5, 10% decrease; flexion to retroversion at S4/S5, 10% decrease).

For the gluteus, retroversion increased CSA and circularity (Table 5, Fig. 2, Fig. 3a). This effect was consistent across levels L5/S1, S1/S2, and max FH, where there was up to a 22% increase from standing or flexion to retroversion (Table 5, Fig. 2a–c; Fig. 3a). However, this effect with retroversion varied at S4/S5 where there was no change in CSA, but increased circularity (Table 5; Fig. 2d–f; Fig. 3b).

**Table 5:** Summary table of significant effects of retroversion posture on gluteus and iliopsoas parameters (P < 0.05, Neuman-Keuls significant). Percent change in parameter is listed, in some cases at a particular level as indicated in the brackets. (n.s. = non significant, − = decrease)

Parameter	From posture	To retroversion for the gluteus (% change)	To retroversion for the iliopsoas (%change)
CSA	Supine	− 19 (S4/S5)	17 (L5/S1)
	Standing	n.s.	n.s.
	Flexion	22 (L5/S1)	15 (L5/S1)
Circularity	Supine	n.s.	− 13 (L5/S1), 12 (Max FH)
	Standing	n.s.	n.s.
	Flexion	20 (Max FH), 24 (S4/S5)	n.s.
Radius	Supine	− 36 (S4/S5)	16 (S1/S2)
	Standing	− 15 (S4/S5)	8 (S1/S2)
	Flexion	− 40 (S4/S5)	15 (S1/S2)
Angle	Supine	10 (S1/S2), − 10 (S4/S5)	3
	Standing	n.s.	n.s.
	Flexion	n.s.	4

Retroversion decreased gluteus radius and increased or decreased gluteus angle at particular levels (Table 5; Fig. 3c,d). At S1/S2 there was up to a 10% increase in angle. At S4/S5 there was up to a 39% decrease in radius, and a 10% decrease in angle.

**Figure 4:**
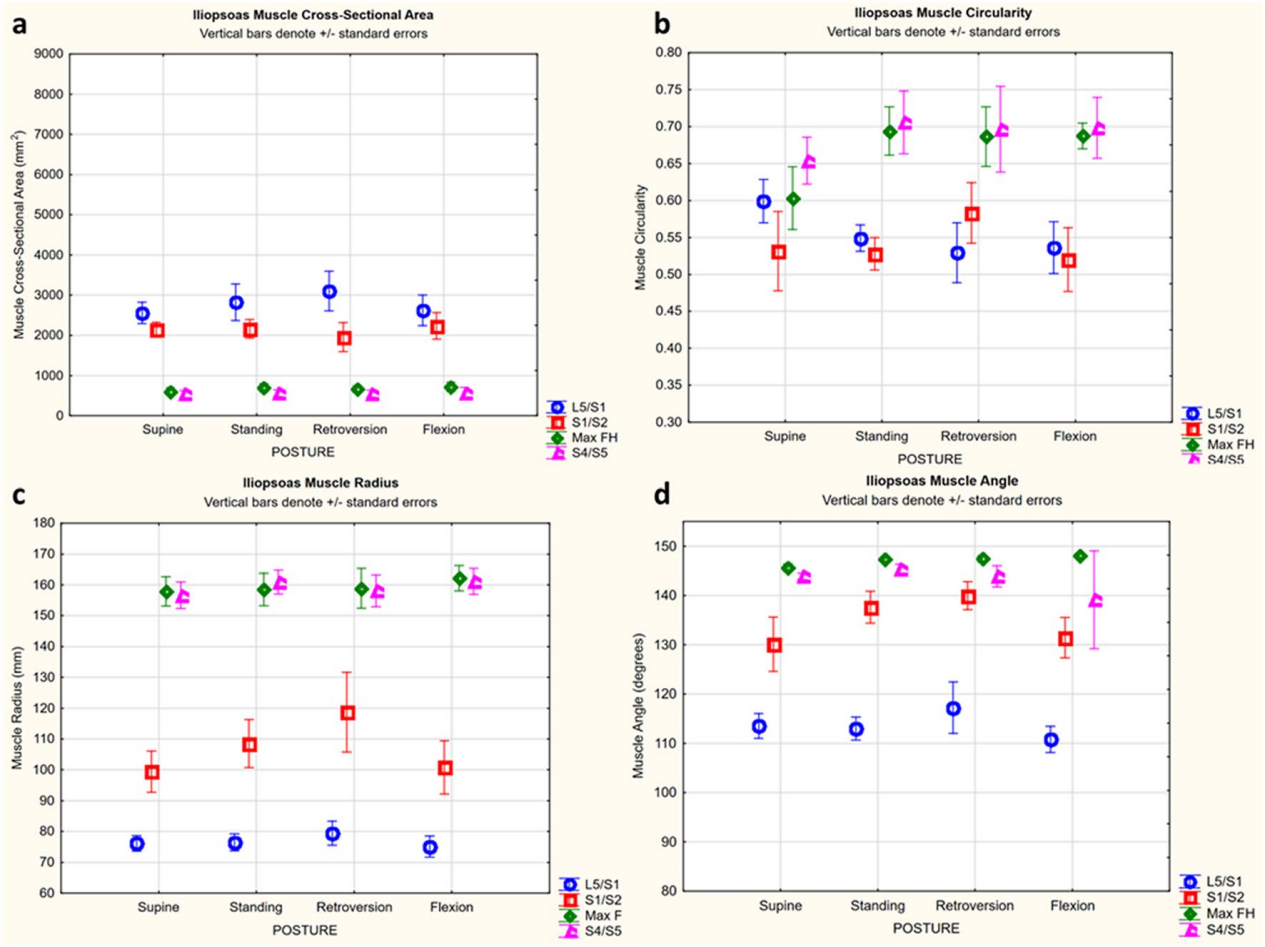
Mean (± SEM) iliopsoas muscle parameter for each posture shown by level. (**a**) CSA: there was a significant effect of level (Newman–Keuls significant: between all levels). At L5/S1 there was also an interaction with posture (Newman–Keuls significant: supine to retroversion, 17% increase; flexion to retroversion, 15% increase). (**b**) Circularity: there was a significant effect and interaction of posture and level (Newman–Keuls significant: L5/S1 supine to flexion, 12% decrease; L5/S1 supine to retroversion, 13% decrease; max FH supine to standing, 13% increase; supine to flexion, 12% increase; supine to retroversion 12% increase). (**c**) Radius: there was a significant effect and interaction of posture and level (Newman–Keuls significant at S1/S2: supine to standing, 8% increase; supine to retroversion, 16% increase; standing to retroversion, 8% increase; flexion to retroversion, 15% increase). (**d**) Angle: there were significant effects of both posture and level (Newman–Keuls significant: between all levels, 5–22% increase; supine to retroversion, 3% increase; standing to flexion, 3% decrease; retroversion to flexion, 4% decrease).

For the iliopsoas, retroversion increased CSA, and increased or decreased circularity at particular levels (Table 5; Fig. 4a,b). For CSA, at L5/S1 there was up to a 17% increase. For circularity, at L5/S1 there was up to a 13% decrease, and at max FH there was up to a 13% increase (Table 5; Fig. 4a).

Retroversion increased iliopsoas radius, and increased or decreased angle at particular levels (Table 5; Fig. 4c,d). For radius, at S1/S2 there was up to a 16% increase. For angle, considering all levels, there was up to a 4% change in angle.

### Secondary objective: pelvic geometry

Retroversion increased PT, decreased SS, and increased L3–S1 LL, but had no effect on PI (Table 6, Supplementary Material Table 7). PT increased up to 68% (16° difference), SS decreased up to 55% (19° difference), and L3–S1 LL increased up to 38% (18° difference).

**Table 6:** Summary table of significant effects of retroversion posture on bony pelvic geometry parameters (P < 0.05, Neuman-Keuls significant). Percent change in parameter and difference are listed. (n.s. = non significant, − = decrease)

Parameter	From posture	To retroversion posture	
		% change	Difference (°)
PT	Supine	53	13
	Standing	28	7
	Flexion	68	16
SS	Supine	− 28	10
	Standing	− 16	5
	Flexion	− 55	19
L3–S1 LL	Supine	n.s.	n.s.
	Standing	n.s.	n.s.
	Flexion	39	18

### Secondary objective: correlations

**Figure 5:**
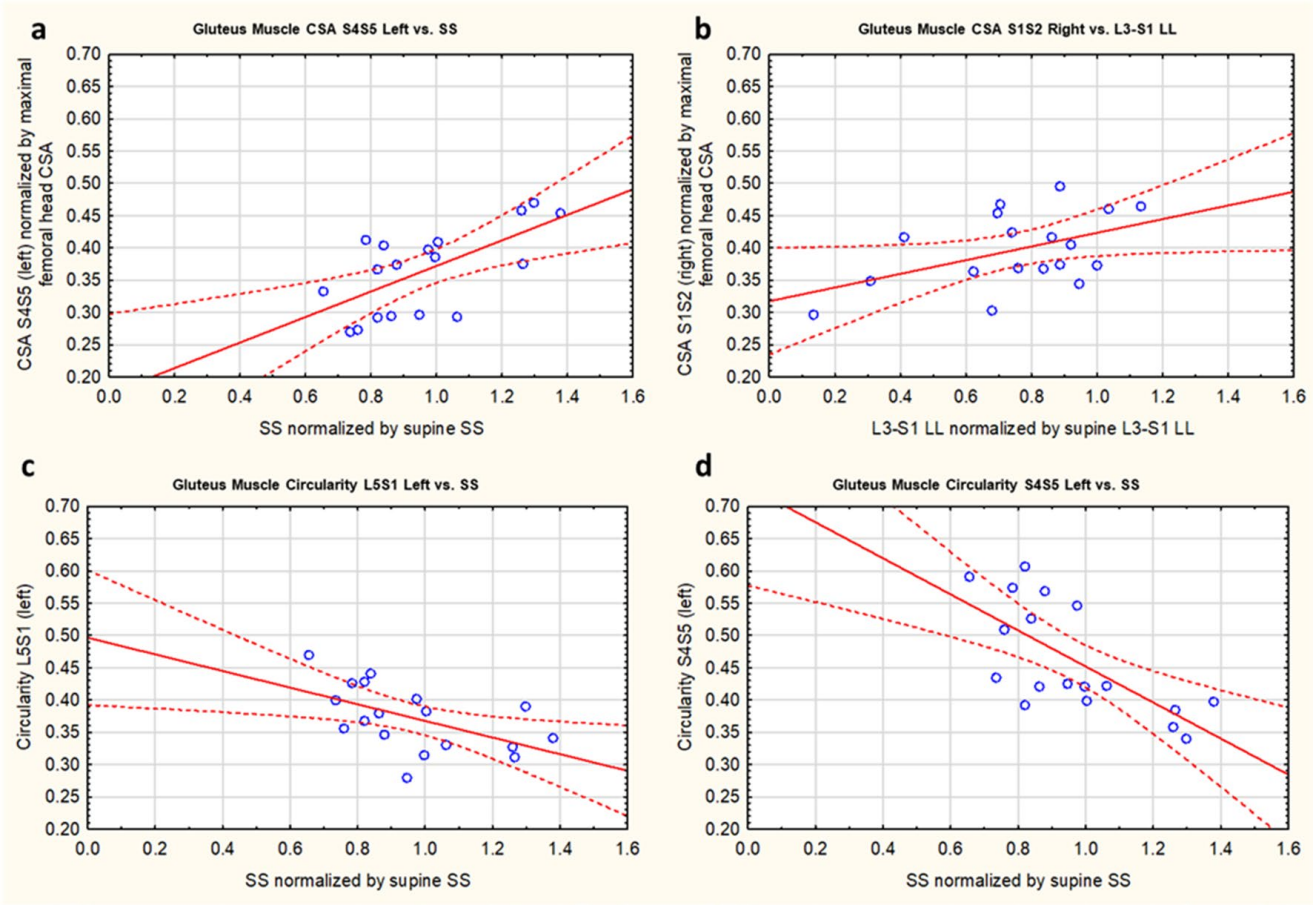
Sample Pearson’s correlation plots of muscle morphometry (CSA and circularity) versus pelvic geometry (SS and L3–S1 LL). CSA was normalized by maximum femoral head cross-sectional area and pelvic geometry (SS and L3–S1 LL) was normalized by supine values.

**Figure 6:**
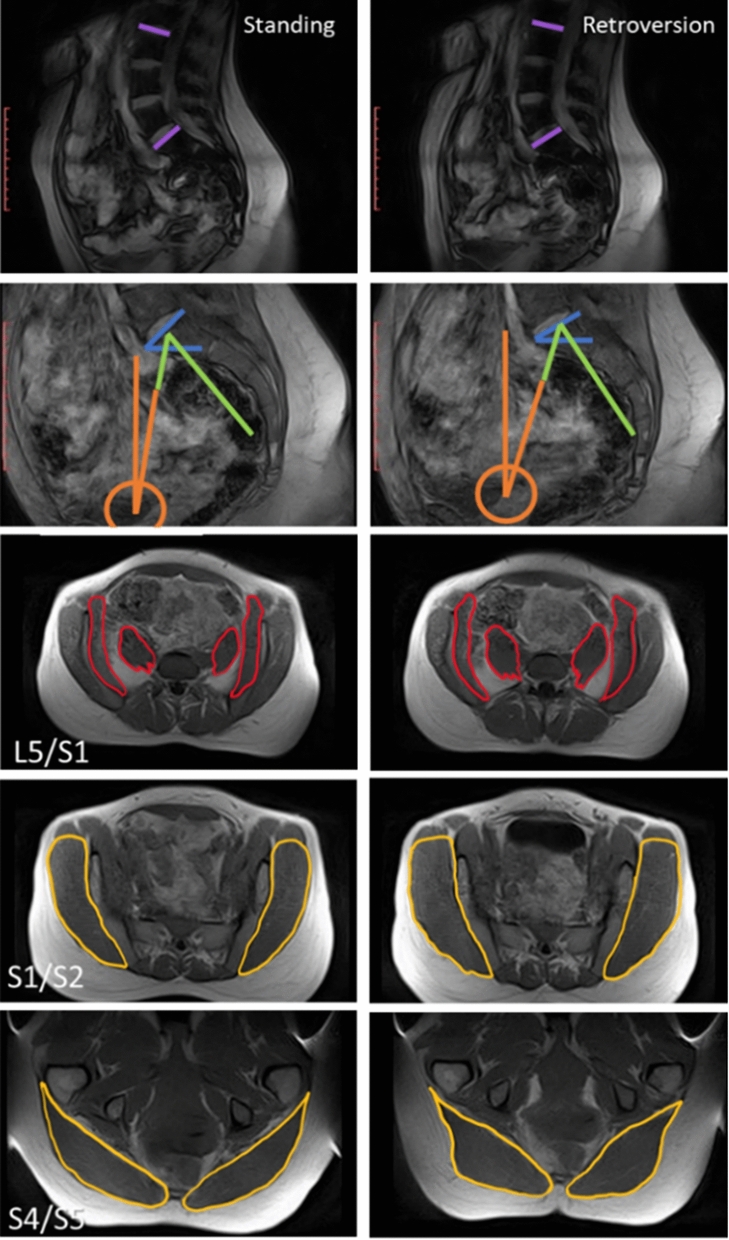
Sample scans illustrating correlations between muscle morphometry and geometry in standing versus retroversion. Standing scans are shown on the left column, and retroversion scans are shown in the right column. Generally, muscle CSA and circularity had significant correlations with PT (positive), SS (negative) and L3–S1 LL (negative) at specific levels. (L3–S1 LL purple, PT orange, PI green, SS blue, iliacus and psoas major red, gluteus yellow).

For the gluteus, there were significant correlations between PT and CSA at S1/S2 on the left (positive). For SS, there were significant correlations with CSA at S4/S5 (positive), and with circularity at L5/S1, max FH, and S4/S5 (negative) for particular sides (Table 7, Figs. 5, 6). For L3–S1 LL there were significant correlations with CSA at S1/S2 and max FH (positive), and with circularity at L5/S1 and S1/S2 (positive) for particular sides.

**Table 7:** Pearson’s correlation (r) of muscle morphometry (CSA and circularity) versus pelvic geometry (PT, SS, and L3–S1 LL).

Muscle	Level	Side	CSA			Circularity		
			PT	SS	LL	PT	SS	LL
Gluteus	L5/S1	Left	0.08	− 0.46	0.15	0.30	− **0.54**	0.11
		Right	− 0.29	− 0.09	0.44	− 0.11	− 0.12	**0.61**
	S1/S2	Left	**0.48**	− 0.13	0.22	0.27	− 0.43	0.25
		Right	0.38	0.07	**0.48**	0.29	− 0.32	**0.52**
	Max FH	Left	0.37	0.25	**0.52**	0.25	− **0.72**	0.21
		Right	0.44	0.10	0.32	0.29	− **0.73**	0.13
	S4/S5	Left	0.05	**0.64**	0.00	0.31	− **0.69**	0.09
		Right	0.13	0.44	0.11	0.37	− **0.52**	0.13

CSA was normalized by maximum femoral head cross-sectional area and pelvic geometry (PT, SS, and LL were normalized by supine values). Bold indicates significant results, P < 0.05.

## Discussion

For the primary objective, this pilot study provides baseline values of parameters in a small group of subjects and illustrates the feasibility and repeatability of imaging both the lumbopelvic muscle morphometry and bony geometry in tandem. This includes generally good repeatability for posture, inter-rater, and intra-rater which is critical as obtaining both muscle and bony geometry measures, simultaneously using upright MRI, has not previously been done and is key to confirm before moving to patient imaging. Overall, having baseline values from pilot healthy subjects, as well as confidence in methodology repeatability and feasibility, is a first step towards designing and performing larger comprehensive studies involving greater number of healthy subjects and patients.

As a secondary objective, this pilot study also explored some effects of pelvic retroversion on lumbopelvic musculature and geometry. This includes demonstrating some preliminary expected trends with retroversion such as muscle widening with shortening, and associated changes in pelvic geometry. Additionally, the differences between supine and upright postures also start to suggest the importance of considering posture specific morphological parameters which may influence downstream biomechanical modeling. Though as a pilot study, some of the strength of the effects may change with future studies with expanded subject numbers, these findings nonetheless help provide a foundation in regard to exploratory effects and implications.

### Repeatability

Repeatability was generally good to excellent for posture, intra- and inter-rater for both muscle and geometry. The few cases of poorer iliopsoas repeatability were likely due to the lower image contrast from the upright MRI’s 0.5 T field strength, combined with signal loss inferiorly, which meant the iliopsoas was harder to differentiate in some individuals. For pelvic geometry, repeatability was generally excellent with only two measures of inter-rater repeatability at 0.79 and 0.82 [ICC(3,1)]. Additionally, the good to excellent PI repeatability across postures is reassuring as PI is a fixed morphological parameter which should not change with posture. Overall, the promising repeatability is key for supporting the feasibility of measuring both lumbopelvic muscle morphometry and pelvic bony geometry in tandem using upright MRI, something that has not previously been done to our knowledge. This helps lay the groundwork for future studies considering both muscle morphometry and bony geometry synchronously in various upright postures, for expanded subject and patient numbers.

### Muscle effects due to posture

With pelvic retroversion, the trends observed in gluteus muscle CSA, circularity and position may be detecting both passive and active muscle changes to achieve a retroverted posture. Previous work has highlighted that the gluteus and hamstring muscles are involved in pelvic retroversion^9,24^. Consequently, the increase in muscle CSA up to 22% from standing or flexion compared to retroversion, and increase in muscle circularity up to 24%, could be representing the muscle’s active engagement to enable this posture. Though muscle length could not be measured due to the upright MRI’s limited field of view, it could be inferred from kinematics that the gluteus muscle would shorten with pelvic retroversion^34,35^. With the muscle shortening from standing or flexion to retroversion, and under the assumption of constant muscle volume, the corresponding increase in muscle CSA and circularity is in line with what could be expected. Though previous studies have shown that gluteus maximus activates with pelvic tilt^9,36,37^, another study found this activation was not significant when compared to neutral^38^. As a result, it may be prudent to speculate that the observed changes in muscle CSA and circularity with retroversion are likely a combination of passive and active muscle engagement.

Additionally, the varying muscle morphometry between supine and standing highlights the importance of considering posture, for example, for biomechanical model development. For the gluteus, there was generally decreasing CSA and circularity from supine to standing by up to 15%. Gluteus position (radius and angle) also changed up to 39% between supine and standing, which can be interpreted to biomechanically represent a moment arm^39^. This could be due to flattening and repositioning of the gluteus muscle, against the scanner table, as an individual is in a supine posture. Similarly, for the iliopsoas, the effect of posture at particular levels on CSA, radius, and angle, in particular between supine and other postures, demonstrated changes up to 22%. Though supine imaging is typically used in biomechanical model development, these differences between standing and supine morphometry start to illustrate, that supine data may not be the most representative of upright postures. This highlights the importance of considering muscle morphometry from different postures in future spinopelvic biomechanical modeling.

### Pelvic geometry effects due to posture

Generally, the pelvic geometry measures followed relationships previously established using radiographic imaging. First, PI did not change with posture which aligns with the accepted view that PI is a fixed parameter for any individual and describes the alignment between the sacrum and the pelvis^9,10,29^. In contrast, PT and SS are functional parameters which changed with posture while following expected trends. Standing postures had an increase in PT (up to 53%)^40^ and a decrease in SS (up to 28%) compared to supine^10^. With pelvic retroversion from standing, PT further increased by up to 28%, and SS decreased by up to 16%^9,10^.

Previous work also established the relationship of PI = PT + SS^9,10,29^. This relationship was observed in this study across all postures and volunteers. The average percent difference between PT + SS and measured PI was only 1.7% (range 0–4.7%) which corresponds to a mean absolute difference of 0.9° (range 0°–2.5°). This provides confidence in using an upright MRI to measure bony pelvic geometric parameters, something that has not been previously done to our knowledge.

Additionally, the influence of PI on compensation and notably the interplay of PI, PT and SS to achieve pelvic retroversion was also observed in this study. Previous work has shown that the ability to retrovert the pelvis, by increasing PT, is limited by PI, in addition to a minimal value of SS of 0°, a horizontal sacral endplate^10,22^. A smaller PI means the pelvis has a more vertical shape, a lower SS and as a result, it is generally observed that these individuals have less capacity to retrovert their pelvis. In comparison, a larger PI means a more horizontal pelvis with a higher SS and larger ability for pelvic retroversion. In this study, when the 2 individuals with the lowest PI (averages of 47° and 50° across all postures) were instructed to retrovert their pelvis, they were only able to achieve a PT of 18° (compared to a standing PT of 7° and 10° respectively). In comparison, the 4 other individuals in this study were able to achieve an average PT of 26° with retroversion (range 24–33°) compared to an average standing PT of 21° (range 15°–26°). Overall, these observations align with accepted trends between PI, PT and SS of previous studies, and supports the feasibility of using an upright MRI for measuring pelvic geometry in varying postures.

### Changing muscle morphometry and bony geometry relationships

The correlations between muscle CSA and circularity with PT, SS, and L3–S1 LL at specific levels helps illustrate the interactions between muscle morphometry and geometry with posture. Notably, from standing to retroversion, the increase in PT and decrease in SS generally correlated to an increase in muscle CSA and circularity. Under the accepted assumption that muscle volume remains constant, the increase in PT and decrease in SS could be corresponding to a shortening of the gluteus muscle which would result in an increasing CSA, which aligns to the observed trend in this study. The additional increase in muscle circularity may be representative of complementary changes in muscle form due to posture. Overall, this supports that body positioning can influence a muscle’s three-dimensional form and positioning, which highlights considering the relationships and interactions between muscle morphometry and bony geometry in biomechanical analysis.

### Clinical considerations

Clinically, it is generally recognized that PT over 30° is associated with poor health quality of life and increased pain^41^. In our current study, asymptomatic individuals were examined, and an average PT of 24° (range 12°–33°) was achieved when volunteers were asked to retrovert their pelvis (compared to average PT of 17° (range 7°–26°) for neutral standing). In the initial pelvic retroversion test, volunteers were instructed not to bend their knees. To consider further mimicking a symptomatic condition, an additional exploratory posture was considered with 3 volunteers where they could bend their knees and were asked again to maximally retrovert their pelvis. With knee bend, an average PT of 32° (range 30°–35°) was achieved compared to an average standing PT of 7.7° (range 2.4°–13.4°). In symptomatic patients, it is observed that knee bend is an additional compensatory mechanism, and when recruited, allows patients to lessen their pelvic retroversion (decrease PT) to maintain a horizontal gaze^21^. However, in our asymptomatic individuals, mimicking a symptomatic condition, it was found that knee bend was additive in allowing further pelvic retroversion (increase PT). Understandably, a key difference is that in symptomatic patients pelvic retroversion helps achieve a horizontal gaze, whereas in an asymptomatic individual horizontal gaze is already achieved. In some sense it seems that in asymptomatic individuals, pelvic retroversion offsets desired horizontal gaze to a certain point where they can still compensate with their upper body. Afterwards, it is only with additional knee bend that further pelvic retroversion can be achieved while still maintaining a horizontal gaze. Overall, this points to the varying pelvic changes between asymptomatic and symptomatic individuals. It also reemphasizes that asymptomatic population findings, such as in this study, even when mimicking symptomatic condition, need thoughtful consideration when pursuing their possible application to a symptomatic population.

### Limitations

Limitations of this pilot study include a small sample size of 6, which was meant to provide a foundation for future studies with expanded volunteer and/or patient numbers. There was also a limited imaging field of view due to the imaging coil which meant that only L3–S1 lumbar lordosis could be measured as opposed to the typically measured L1–S1 lumbar lordosis, and that muscle length could not be measured. It also meant there was signal loss, and some image artifacts in some muscles in the lower levels and when at the edge of the images, which could make segmentation more challenging and may have resulted in lower repeatability values. Additionally, due to the 0.5 T field strength of the open MR, individual muscle fascicles could not be differentiated and likewise muscle fascicle pennation angle could not be observed. As a result, the CSA measured would not be considered physiological nor anatomical muscle CSA. Moreover, muscle activation was not controlled as volunteers were simply asked to assume and hold postures as naturally as possible.

## Conclusion

This pilot study demonstrates that lumbopelvic muscle morphometry and bony geometry can be measured synchronously in upright and retroverted postures in asymptomatic individuals. For the primary objective, this study provides baseline values of parameters in a pilot group and also illustrates feasibility and good repeatability of imaging both the lumbopelvic muscle morphometry and bony geometry in tandem. As this synchronous imaging has not been previously performed in standing postures using an upright MRI, this is critical groundwork for enabling the design and performance of larger studies involving greater number of healthy subjects as well as future ASD patient imaging. For the secondary objective exploring retroversion and other postures, the significant effects, interactions, and correlations observed between muscle morphometry and bony geometry measures also confirms some expected trends such as muscle narrowing with elongation. Overall, this pilot study presents a method for starting to understand the importance of musculoskeletal interactions between the spine, pelvis, and associated musculature in sagittal compensatory mechanisms for future biomechanical modeling and clinical study.

## Supplementary Information

Supplementary Information.

## Data Availability

The datasets generated and analyzed during the current study are not publicly available due to ongoing research with the datasets in our research group, but are available from the corresponding author on reasonable request.
