# Supplementary material for: Synchronous imaging of pelvic geometry and muscle morphometry: a pilot study of pelvic retroversion using upright MRI
Source: Sci Rep. 2021 Oct 11;11:20127. doi: 10.1038/s41598-021-99305-w (PMC8505414; doi:10.1038/s41598-021-99305-w)
Supplement: Supplementary file 1 — Supplementary Information. [file 41598_2021_99305_MOESM1_ESM.pdf]

Synchronous Imaging of Pelvic Geometry and Muscle Morphometry – A Pilot Study of Pelvic Retroversion Using Upright MRI

Noor Shaikh [1,2,3], Honglin Zhang [4], Stephen H. M. Brown [5], Hamza Lari [6], Oliver Lasry [2,7], John Street [2,7], David R. Wilson [2,4,7], Thomas Oxland [2,3,7]

[1]School of Biomedical Engineering, University of British Columbia, Vancouver, Canada

[2]ICORD, University of British Columbia, Vancouver, Canada

[3]Department of Mechanical Engineering, University of British Columbia, Vancouver, Canada

[4]Centre for Hip Health and Mobility, University of British Columbia, Vancouver, Canada

[5]Department of Human Health and Nutritional Sciences, University of Guelph, Guelph, Canada

[6]Department of Radiology, Vancouver General Hospital, Vancouver, Canada

[7]Department of Orthopaedics, University of British Columbia, Vancouver, Canada

Supplementary Material

Table 1: Posture repeatability of muscle CSA in the re-scanned volunteers using intra-class correlation coefficients ICC(3, 1) and mean absolute difference (MAD) between posture repeats, in comparison with MAD between volunteers.

Posture	Gluteus					Iliopsoas				
	ICC (3,1)	MAD of CSA between posture repeats (mm^2)	MAD as % of mean CSA (%)	MAD of CSA between volunteers (mm^2)	MAD as % of mean CSA (%)	ICC (3,1)	MAD of CSA between posture repeats (mm^2)	MAD as % of mean CSA (%)	MAD of CSA between volunteers (mm^2)	MAD as % of mean CSA (%)
Supine	0.94	548	8	--	--	0.91	515	29	--	--
Standing	0.93	490	10	1541	32	0.90	267	18	543	38
Retroversion	0.86	667	13	1375	27	0.95	289	18	677	42
Flexion	0.87	759	16	1350	28	0.97	191	13	558	38

Table 2: Posture repeatability of all pelvic geometric parameters (PT, PI, SS, LL) in the re-scanned volunteers using intra-class correlation coefficients ICC(3, 1) and mean absolute difference (MAD) between posture repeats, in comparison with MAD between volunteers.

Posture	ICC (3,1)	MAD of parameters between posture repeats (°)	MAD as % of parameter mean (%)	MAD of parameters between volunteers (°)	MAD as % of parameter mean (%)
Supine	0.99	1	--	--	--
Standing	0.98	4	9	9	22
Retroversion	0.97	2	6	7	19
Flexion	0.94	6	17	13	34

Table 3: Intra-rater repeatability of muscle CSA with three raters using intra-class correlation coefficients, ICC(3, 1)

Level	Side	Rater 1		Rater 2		Rater 3	
		Gluteus	Iliopsoas	Gluteus	Iliopsoas	Gluteus	Iliopsoas
L5/S1	Right	0.95	0.95	0.90	0.84	0.89	0.91
	Left	0.98	0.95	0.83	0.93	0.94	0.92
S1/S2	Right	0.98	0.84	0.99	0.62	0.95	0.83
	Left	0.96	0.77	0.96	0.87	0.97	0.84
Max FH	Right	0.96	0.76	0.85	0.81	0.91	0.78
	Left	0.95	0.87	0.92	0.89	0.90	0.91
S4/S5	Right	0.86	0.79	0.86	0.81	0.85	0.68
	Left	0.82	0.85	0.89	0.86	0.89	0.89

Table 4: Intra-rater repeatability of pelvic geometric parameters with three raters using intra-class correlation coefficients, ICC(3, 1)

Parameter	Rater 1	Rater 2	Rater 3
PT	0.99	0.91	0.99
PI	0.95	0.76	0.79
SS	0.99	0.95	0.96
LL	0.99	0.93	0.88

Table 5: Inter-rater repeatability of muscle CSA with three raters using intra-class correlation coefficients, ICC(3, 1)

Level	Side	Muscle	
		Gluteus	Iliopsoas
L5/S1	Right	0.92	0.79
	Left	0.93	0.89
S1/S2	Right	0.94	0.55
	Left	0.96	0.72
Max FH	Right	0.86	0.65
	Left	0.90	0.87
S4/S5	Right	0.77	0.54
	Left	0.76	0.66

Table 6: Inter-rater repeatability of pelvic geometry measures with three raters using intra-class correlation coefficients, ICC(3, 1).

Parameter	ICC (3,1)
PT	0.94
PI	0.79
SS	0.93
LL	0.82

Table 7: Pelvic geometric parameter means and standard error for each posture. Significant results from Newman–Keuls post-hoc are indicated by symbols in the legend.

Posture	PT		SS		PI		LL	
	Mean (°)	Standard Error (°)	Mean (°)	Standard Error (°)	Mean (°)	Standard Error (°)	Mean (°)	Standard Error (°)
Supine	11*	2	44 ^, **, ++	1	54	3	53 ***	3
Standing	17 #, +	3	40 ^, ##, $$	2	56	3	46 ###	4
Retroversion	24*, #, $	2	34 **, ##, ^^	2	57	2	46 +++	5
Flexion	8 +, $	4	53 ++, $$, ^^	3	55	3	28 ***, ###, +++	6

PT: * = significant between supine to retroversion, # = significant between standing to retroversion, + = significant between standing to flexion, $ = significant between retroversion to flexion
SS: ^ = significant between supine to standing, ** = significant between supine to retroversion, ## = significant between standing to retroversion, ++ = significant between supine to flexion, $$ = significant between standing to flexion, ^^ = significant between retroversion to flexion
LL: *** = significant between supine to flexion, ### = significant between standing to flexion, +++ = significant between retroversion to flexion
PI: no significant differences between postures
